# Increased expression of EHF contributes to thyroid tumorigenesis through transcriptionally regulating *HER2* and *HER3*

**DOI:** 10.18632/oncotarget.11154

**Published:** 2016-08-09

**Authors:** Yanyan Lv, Fang Sui, Jingjing Ma, Xiaojuan Ren, Qi Yang, Yanfang Zhang, Haixia Guan, Bingyin Shi, Peng Hou, Meiju Ji

**Affiliations:** ^1^ Department of Endocrinology, The First Affiliated Hospital of Xi'an Jiaotong University, Xi'an 710061, P.R. China; ^2^ Department of Rheumatology, Xi'an No 5 Hospital, Xi'an 710082, P.R. China; ^3^ Key Laboratory for Tumor Precision Medicine of Shaanxi Province, The First Affiliated Hospital of Xi'an Jiaotong University, Xi'an 710061, P.R. China; ^4^ Department of Endocrinology and Metabolism, The First Affiliated Hospital of China Medical University, Shenyang 110001, P.R. China; ^5^ Center for Translational Medicine, The First Affiliated Hospital of Xi'an Jiaotong University, Xi'an 710061, P.R. China

**Keywords:** thyroid cancer, EHF, PI3K/Akt pathway, MAPK pathway, HER family of receptor tyrosine kinases

## Abstract

E26 transformation-specific (ETS) transcription factor EHF plays a tumor suppressor role in prostate cancer and esophageal squamous cell carcinoma (ESCC), whereas it is overexpressed and may act as an oncogene in ovarian and mammary cancers. However, its biological role in thyroid cancer remains totally unknown. The aim of this study was to explore the biological functions of EHF and its potential as a therapeutic target in thyroid cancer. Using quantitative RT-PCR (qRT-PCR) assay, we evaluated mRNA expression of *EHF* in a cohort of primary papillary thyroid cancers (PTCs) and matched non-cancerous thyroid tissues. The functions of knockdown and ectopic expression of *EHF* in thyroid cancer cells were determine by a series of *in vitro* and *in vivo* experiments. Moreover, dual-luciferase reporter and chromatin immunoprecipitation (ChIP) assays were performed to identify its downstream targets. Our data showed that *EHF* expression was significantly increased in PTCs compared with matched non-cancerous thyroid tissues. EHF knockdown significantly inhibited thyroid cancer cell proliferation, colony formation, migration, invasion and tumorigenic potential in nude mice and induced cell cycle arrested and apoptosis by modulating the PI3K/Akt and MAPK/Erk signaling pathways. On the other hand, ectopic expression of *EHF* in thyroid cancer cells notably promoted cell growth and invasiveness. Importantly, EHF was identified as a new transcription factor for *HER2* and *HER3*, contributing to thyroid tumorigenesis. Altogether, our findings suggest that *EHF* is a novel functional oncogene in thyroid cancer by transcriptionally regulating *HER2* and *HER3*, and may represent a potential therapeutic target for this cancer.

## INTRODUCTION

Thyroid cancer, the most common malignancy in endocrine system, has rapidly increased 3-fold over the past 30 years [1–3]. The histopathology of thyroid cancer is diverse, including papillary thyroid cancer (PTC), follicular thyroid cancer (FTC), poorly differentiated thyroid cancer (PDTC), anaplastic thyroid cancer (ATC) and medullary thyroid cancer (MTC). About 80% of all thyroid cancers are PTCs, which are usually benign and curable. The overall 5-year relative survival rate for PTCs is over 95% [4]; however, occasionally they dedifferentiate into more aggressive and lethal thyroid cancers. We thus need a better understanding of molecular mechanisms underlying thyroid tumorigenesis.

The ETS (E26 transformation-specific) transcription family includes 29 genes in humans, such as ELF, ELG, ERF, ERG, ER71, ESE, ETS, PDEF, PEA3, SPI, TCF and TEL [5]. All members of this family contain a highly conserved ETS domain. This domain folds into a winged helix-turn-helix structure and binds to DNA sequence with a core GGAA motif [6]. This family is present throughout the body and is involved in a wide variety of biological functions such as the regulation of cellular differentiation, cell proliferation, cell cycle, survival and invasiveness [7–9].

EHF (also known as ESE-3) belongs to an ETS transcription factor subfamily characterized by epithelial cell-specific expression [10]. There are some evidences showing that EHF plays an important role in the regulation of epithelial proliferation and differentiation, and its aberrant expression may contribute to oncogenesis of epithelium-derived tumors [10–12]. EHF has been suggested to function as an oncosuppressor in prostate cancer and esophageal squamous cell carcinoma (ESCC) [11, 13, 14], and be frequently silenced by promoter methylation [13]. On the other hand, it is found to be overexpressed in ovarian and mammary cancers [15–17] and be associated with poor survival in ovarian cancer [15, 16]. However, the biological roles of EHF in thyroid cancer is largely unknown.

In this study, we demonstrated *EHF* overexpression in primary PTCs. EHF knockdown significantly reduced *in vitro* and *in vivo* oncogenic potential of thyroid cancer cells by regulating HER family of receptor tyrosine kinases. Moreover, EHF overexpression dramatically enhanced the growth and invasive abilities of cancer cells, further supporting its oncogenic function.

## RESULTS

### Increased expression of EHF promotes thyroid cancer cell growth

**Figure 1:**
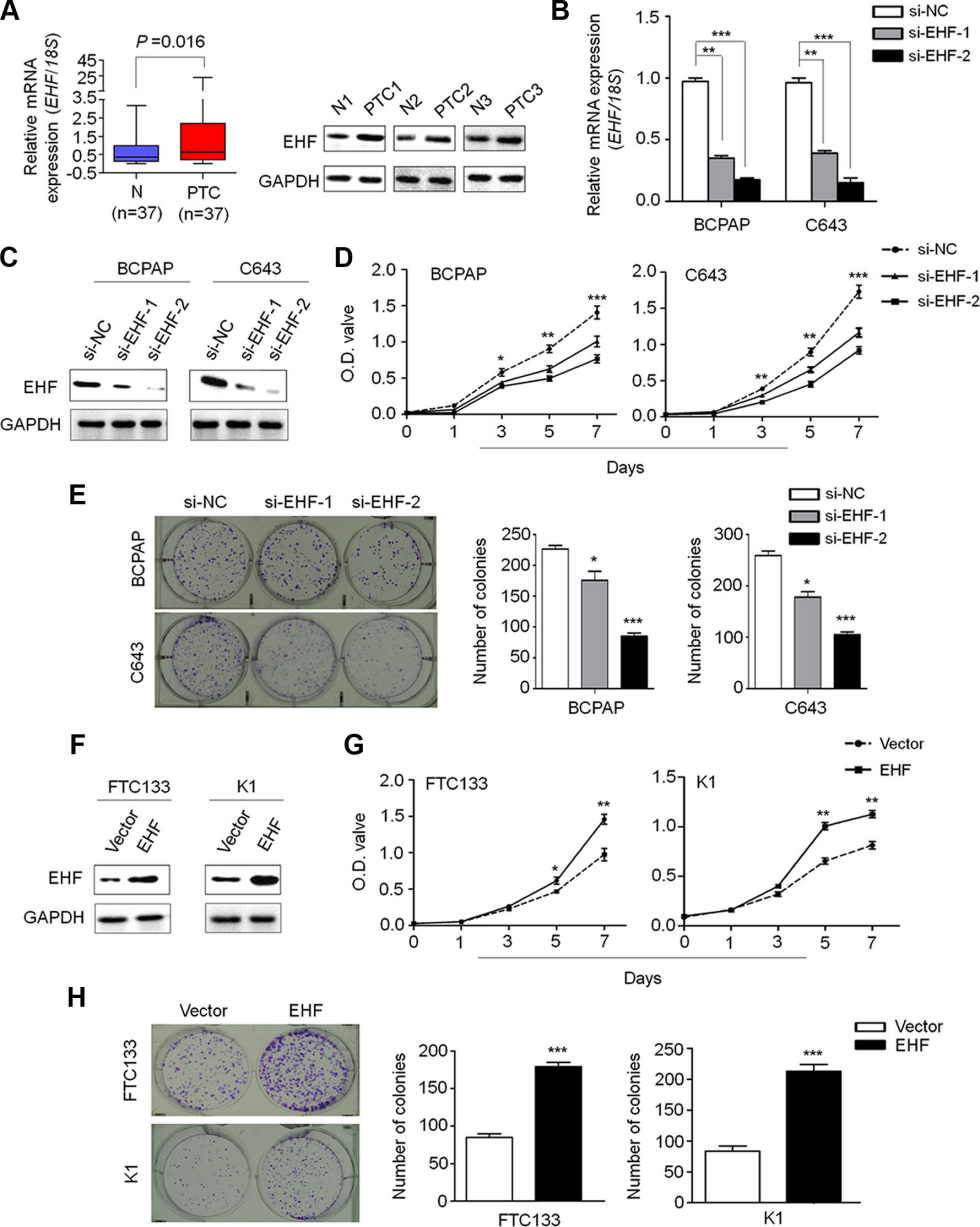
Increased expression of EHF promotes thyroid cancer cell proliferation and colony formation qRT-PCR (**A**, left panel) and western blot (A, right panel) assays were performed to evaluate EHF expression in primary PTCs and their matched non-cancerous thyroid tissues (N). *EHF* expression was normalized with *18S* rRNA levels. GAPDH was used as loading control in western blot assay. Knockdown of *EHF* mRNA (**B**) and protein (**C**) by using two different siRNAs (si-EHF-1 and si-EHF-2) in BCPAP and C643 cells was evidenced by qRT-PCR and western blot assays, respectively. *18S* rRNA was used as a normalized control for qRT-PCR assay. GAPDH was used as loading control in western blot assay. The data were presented as mean ± SE. (**D**) EHF knockdown significantly inhibited thyroid cancer cell proliferation. The data were presented as mean ± SE. (**E**) EHF knockdown significantly inhibited colony formation of thyroid cancer cells. Left panel shows the representative images of colony formation in cells transfected with the indicated siRNAs. Quantitative analysis of colony numbers is shown in right panel. The data were presented as mean ± SE. (**F**) EHF expression levels were increased after transfection with *EHF* expression plasmid in FTC133 and K1 cells. Ectopic expression of EHF significantly enhanced cell proliferation (**G**) and colony formation (**H**) in these two cell lines. The data were presented as mean ± SE. Statistically significant differences were indicated: *P < 0.05; **P < 0.01; ***P < 0.001.

We first examined EHF expression in a cohort of primary PTCs and matched non-cancerous thyroid tissues (control subjects) at mRNA levels using qRT-PCR assay and at protein levels using western blot analysis. As shown in Figure 1A (left panel), mRNA expression of *EHF* was up-regulated in primary PTCs compared with control subjects (*P* = 0.016), which was further supported by the results of western blot (Figure 1A, right panel). We also investigated mRNA expression of *EHF* in normal thyroid tissues and different subtypes of PTCs using The Cancer Genome Atlas (TCGA) dataset. The results showed that *EHF* expression in normal thyroid tissues was significantly lower than that in tall-cell variant of PTCs (TCV-PTCs) and conventional PTCs (CPTCs), but higher than that in follicular variant of PTCs (FVPTCs). Moreover, we also found that EHF expression was the highest in TCV-PTCs, second in CPTCs and least in FVPTCs (Supplementary Figure S1).

To explore the biological roles of EHF in thyroid tumorigenesis, we first chosen thyroid cancer cell lines BCPAP and C643 with high basal levels of EHF for knockdown experiment, and FTC133 and K1 with low basal levels of EHF for overexpression experiment (data not shown). Next, we tested the growth-suppressive effect by knocking down EHF expression in BCPAP and C643 cells using siRNA approach. EHF knockdown by two different *EHF* siRNAs (si-EHF-1 and si-EHF-2) was validated by qRT-PCR (Figure 1B) and western blot (Figure 1C). EHF knockdown significantly inhibited cell proliferation (Figure 1D) and colony forming ability in monolayer culture (Figure 1E) compared to the control. On the other hand, ectopic expression of EHF in FTC133 and K1 cells (Figure 1F) significantly promoted cell proliferation as compared with empty vector (Figure 1G and 1H). Altogether, these data suggest the oncogenic function of EHF in thyroid cancer.

### EHF knockdown induces thyroid cancer cell cycle arrest and apoptosis

**Figure 2:**
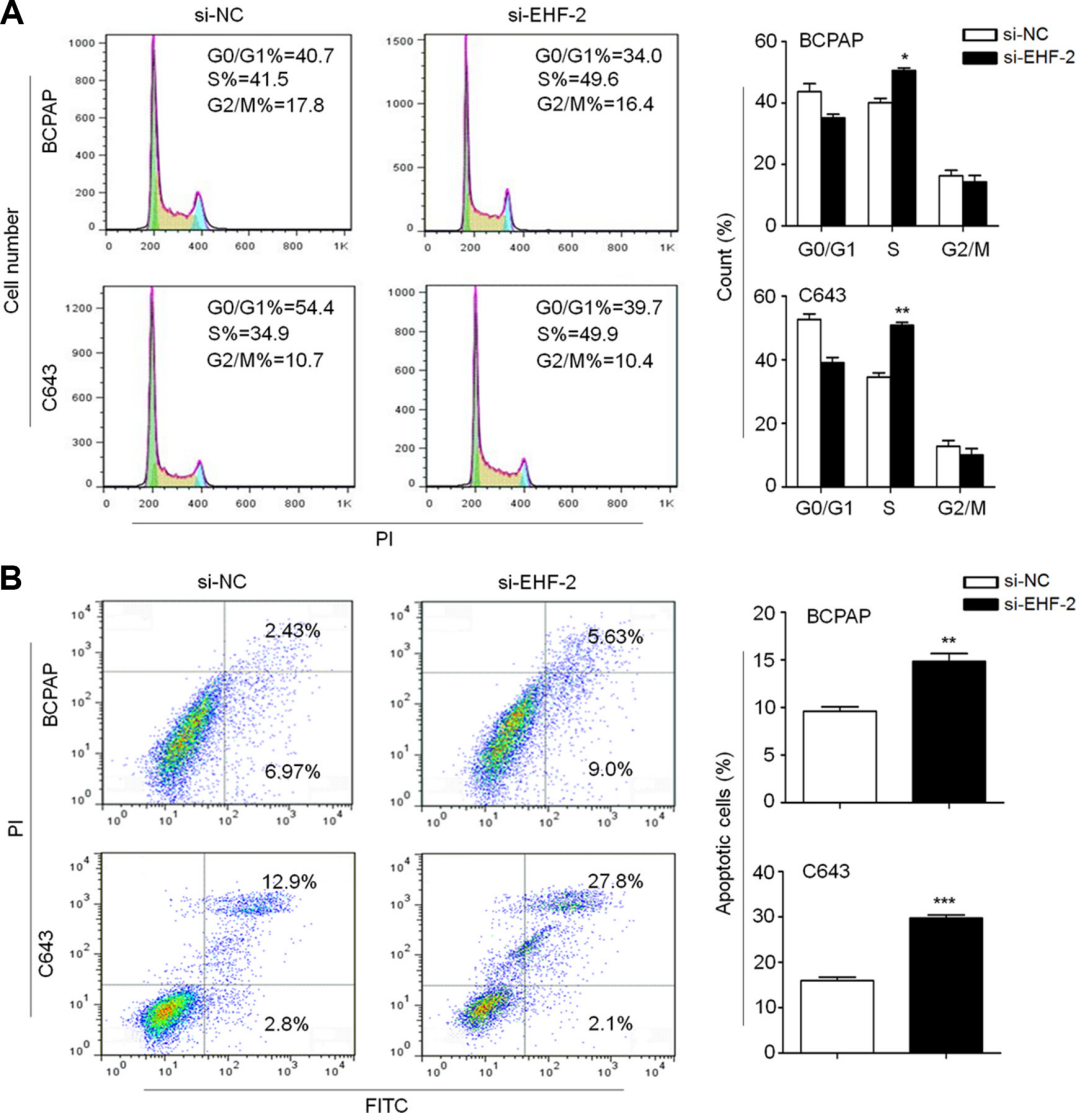
EHF knockdown induces cell cycle arrest and apoptosis in thyroid cancer cells (**A**) BCPAP and C643 cells were transiently transfected with si-EHF-2 or si-NC. DNA content was measured by flow cytometry to determine cell cycle fractions. The fraction of cells in each cell cycle phase was indicated in the figures. (**B**) Apoptotic cells including early and late apoptotic cells were measured 48 h after transfection by flow cytometry analysis of Annexin V-FITC/PI double-labelled cells. The data were presented as mean ± SE of values from three independent experiments. Statistically significant differences were indicated: *P < 0.05; **P < 0.01; ***P < 0.001.

Next, we evaluated the impact of EHF depletion on cell cycle distributions and apoptosis in thyroid cancer cells. As shown in Figure 2A, cell cycle arrest at S phage was observed in si-EHF-2 transfected cells compared to control cells (Figure 2A). The percentage of S-phase cells was increased from 40.1 ± 1.5% to 50.5 ± 0.9% in BCPAP cells (*P* = 0.01) and from 34.6 ± 1.3% to 50.8 ± 0.9% in C643 cells (*P* = 0.003), respectively. In addition, si-EHF-2 transfection caused an increase in the number of apoptotic cells compared with control cells (Figure 2B). The percentage of apoptotic cells was increased from 9.6 ± 0.8% to 14.8 ± 1.4% in BCPAP cells (*P* = 0.005) and from 8.6 ± 2.3% to 17.5 ± 1.4% in C643 cells (*P* = 0.0003), respectively.

### EHF knockdown inhibits tumor growth in nude mice

**Figure 3:**
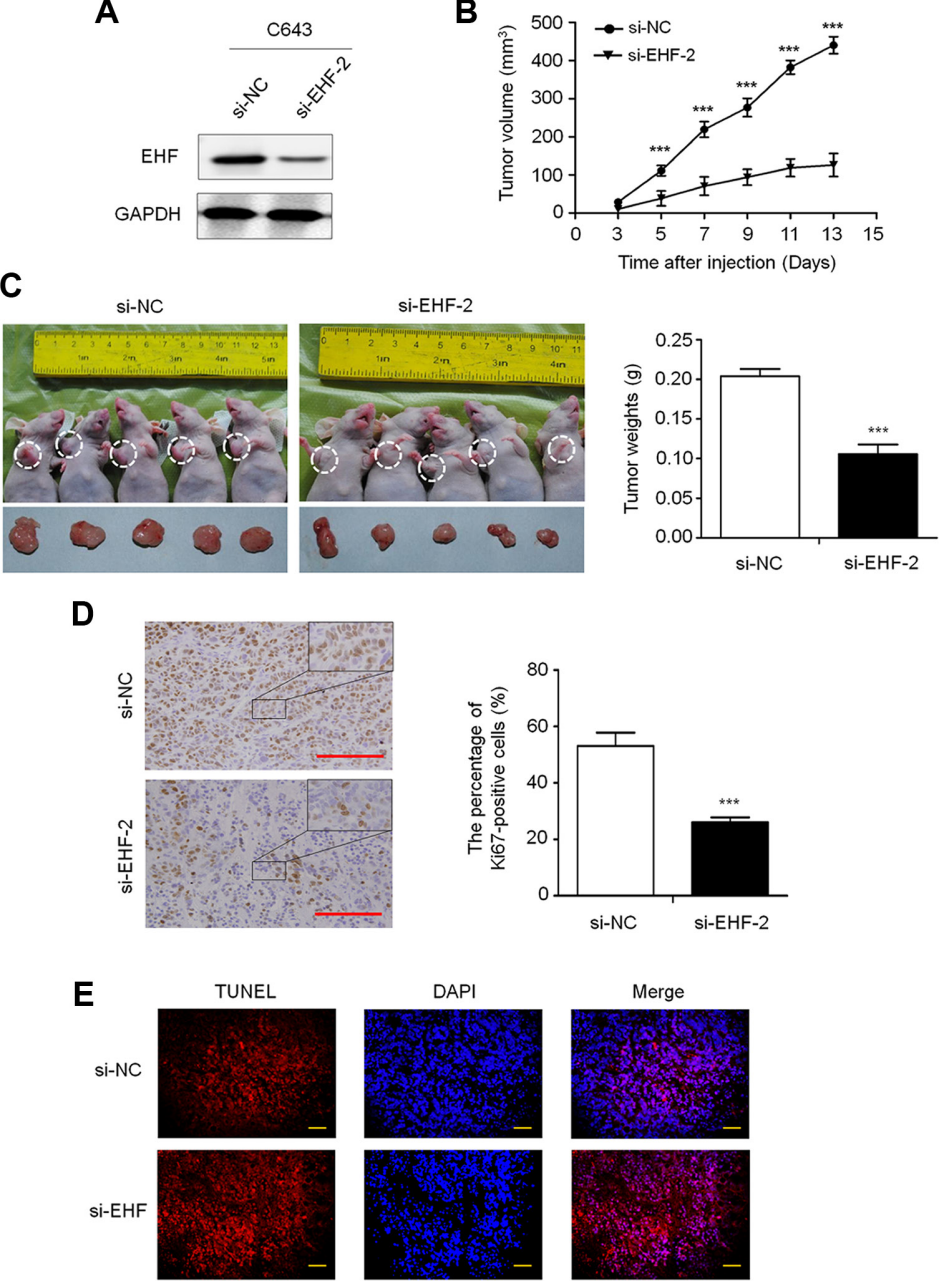
EHF knockdown inhibits the xenograft tumor growth (**A**) Knockdown efficiency of EHF in C643 cells by si-EHF-2 transfection was demonstrated by western blot. (**B**) Subcutaneous tumor growth curve of si-EHF-2 transfected C643 cells in nude mice was compared with si-NC transfected cells. The si-EHF-2 group showed a retarded tumor growth compared to the si-NC group. Data are shown as mean ± SD (*n* =5/group). (**C**) A representative picture for tumor growth of cells transfected with the indicated siRNA in nude mice (left panel). Histogram represents mean of tumor weight from the si-EHF-2 and si-NC groups (right panel). Data are shown as mean ± SD (*n* = 5/group). (**D**) Shown is representative Ki-67 staining of xenograft tumors from the si-EHF-2 and si-NC groups (left panels). Histogram represents mean ± SE of the percentage of Ki-67-positive cells from 5 microscopic fields in each group (right panel). Scale bar, 200 μm. (**E**) The TUNEL (red) and DAPI (blue) staining in the xenograft tumors from si-EHF-2 and si-NC groups. Scale bar, 50 μm. Statistically significant differences were indicated: ***P < 0.001.

To further investigate the *in vivo* tumorigenic ability of EHF, we knocked down the expression of EHF in C643 cells using siRNA approach (Figure 3A). The si-EHF-2-C643 and control (si-NC-C643) cells were inoculated subcutaneously in nude mice. Tumor induced by si-EHF-2-C643 cells showed significantly longer latency and smaller mean tumor volume than tumors induced by si-NC-C643 cells (Figure 3B). At the end of experiments, xenograft tumors were isolated and weighed. The mean tumor weight of the si-EHF-2-C643 group was significantly less compared with control group (*P* < 0.001; Figure 3C). To assess the proliferation index in the xenograft tumor, Ki-67 staining was performed on paraffin-embedded tumor sections. The percentage of Ki-67 positive tumor cells was significantly decreased in si-EHF-2-C643 group compared with control group (*P* = 0.009; Figure 3D). In addition, the TUNEL assay showed that the number of apoptotic cells was reduced upon EHF knockdown in xenograft tumors compared to the control (Figure 3E). These observations further support that *EHF* is a functional oncogene in thyroid cancer.

### EHF knockdown inhibits thyroid cancer cell migration and invasion

**Figure 4:**
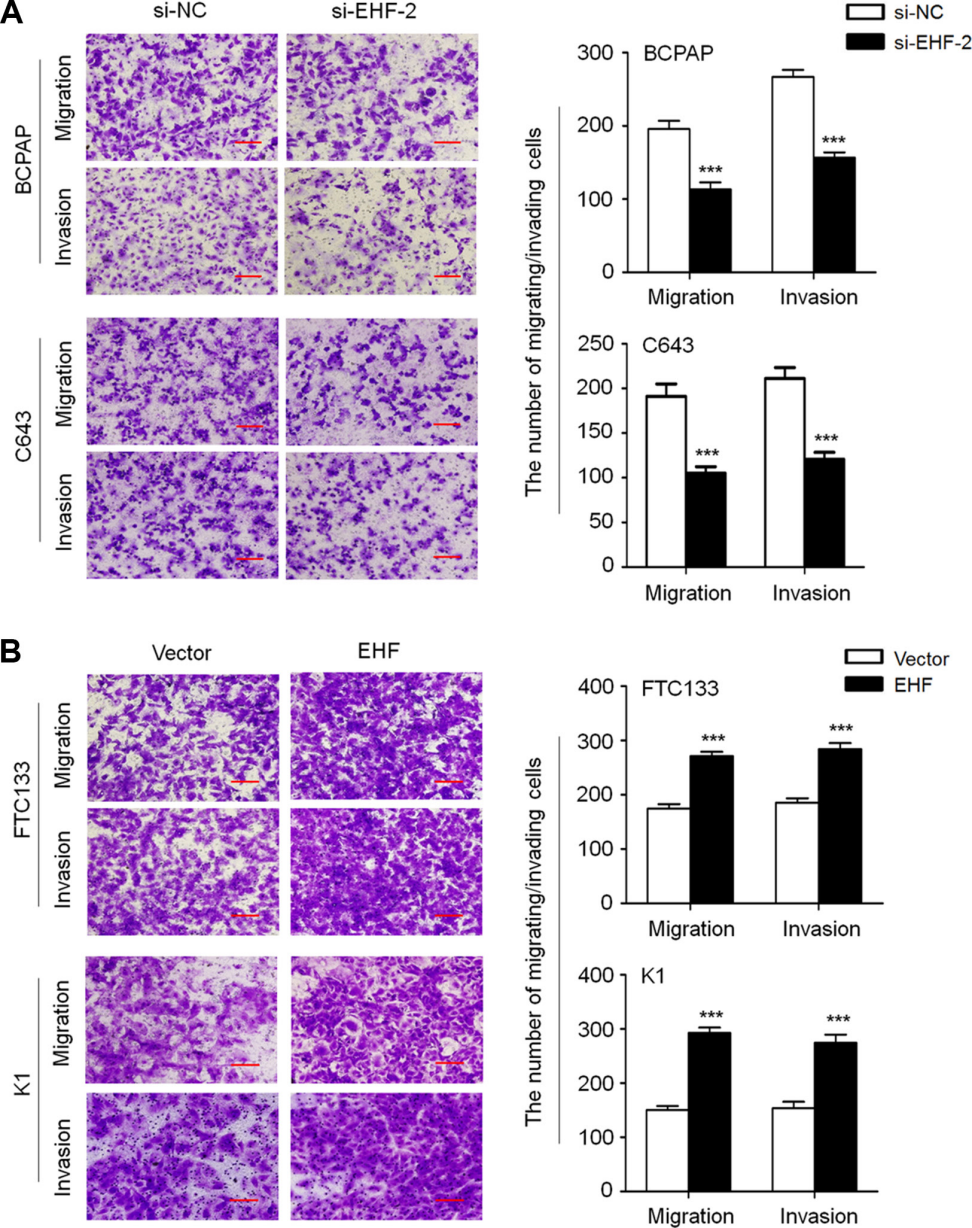
EHF knockdown inhibits thyroid cancer cell migration and invasion (**A**) EHF knockdown suppressed cell migration and invasion in BCPAP and C643 cells. (**B**) Ectopic expression of EHF promoted cell migration and invasion in FTC133 and K1 cells. The representative images of migrated/invaded cells (left panels). Histograms, corresponding to upper panels, show means ± SE of cell numbers from three independent assays (right panels). Statistically significant differences were indicated: ***P < 0.001.

We also tested the impact of altered EHF expression on cell migration and invasion. The results showed that the number of migrated/invaded cells was dramatically reduced in the si-EHF-2 transfected cells compared to control cells (Figure 4A). On the other hand, EHF overexpression significantly enhanced cell migration/invasion abilities in FTC133 and K1 cells (Figure 4B). These data suggest that there is a strong link between increased expression of EHF and metastatic phenotypes in thyroid cancer.

### EHF transcriptionally regulates *HER2* and *HER3* expression in thyroid cancer

**Figure 5:**
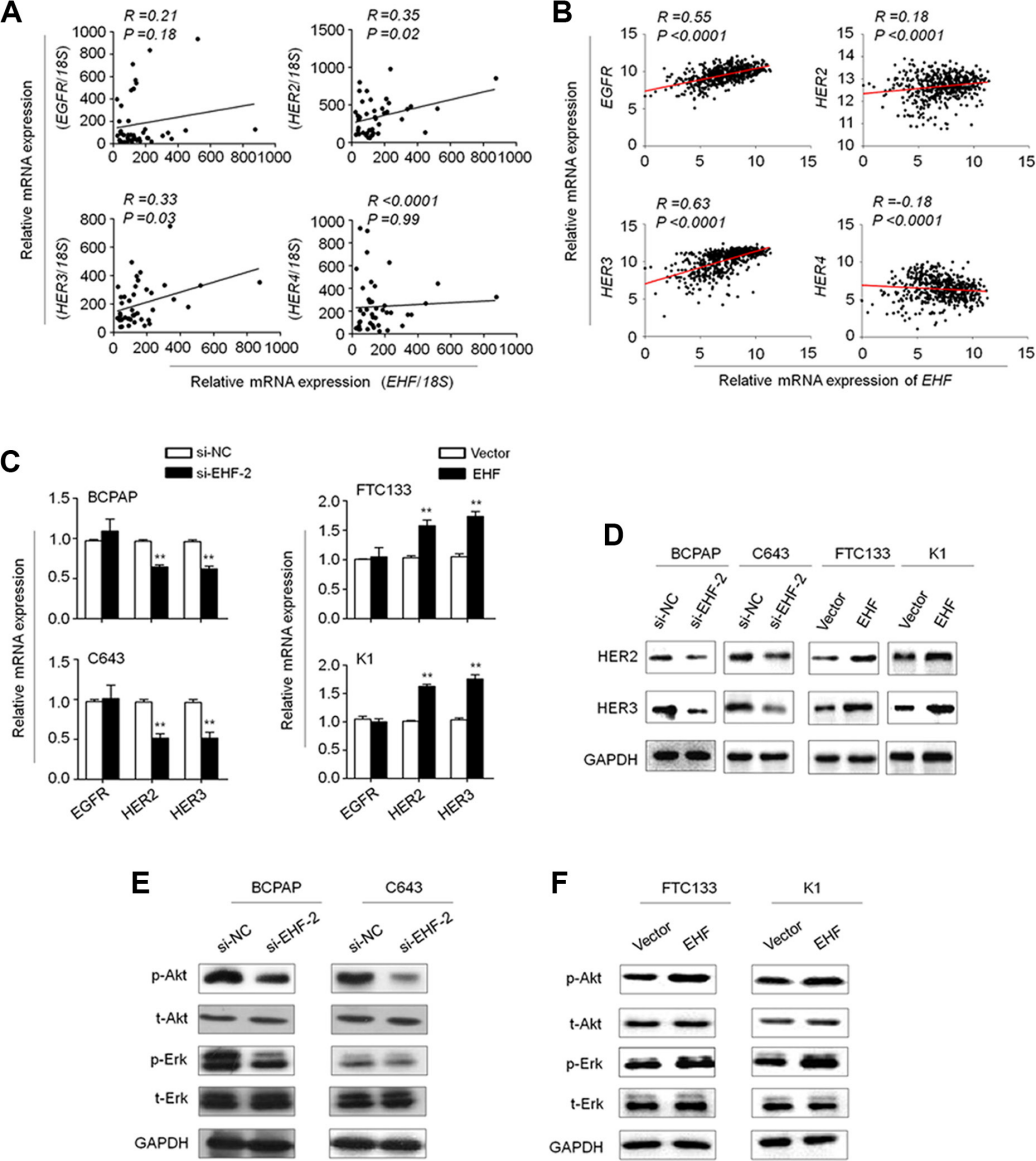
EHF regulates the expression of *HER2* and *HER3* and the activities of their downstream signaling pathways in thyroid cancer The relationships between with mRNA expression of *EHF* and HER receptors (EGFR, HER2, HER3 and HER4) in primary PTCs from our cohort (**A**, *n* =37) and TCGA cohort (**B**, *n* = 513). (**C**) qRT-PCR assay was performed to investigate the effect of EHF knockdown on the expression of *EGFR*, *HER2* and *HER3*. Expression levels of these genes were normalized with *18S* rRNA levels. Data were presented as mean ± SE. (**D**) Western blot assay was used to confirm the effect of EHF knockdown or overexpression on the expression of HER2 and HER3. (**E**, **F**) The lysates from the indicated cells were subjected to western blot assays. The antibodies against phospho-Akt (p-Akt), total Akt (t-Akt), phospho-Erk (p-Erk) and total Erk (t-Erk) were used to determine the effect of EHF knockdown or overexpression on the activities of the PI3K/Akt and MAPK/Erk pathways. GAPDH was used as a loading control.

The HER/ErbB family of receptors including EGFR (HER1/ErbB1), HER2 (ErbB2), HER3 (ErbB3) and HER4 (ErbB4) plays a critical role in tumorigenesis including thyroid cancer [18–20]. Using MatInspector online software (www.genomatix.de/online_help/help_matinspector/matinspector_help), we found several potential ETS binding sites (EBS, 5′-GGAA/T-3′) in their promoter regions. We thus speculate that oncogenic role of EHF in thyroid tumorigenesis may be associated with the activation of HER family. As shown in Figure 5A, *EHF* expression was significantly positively correlated with the expression of *HER2* (*R* = 0.35; *P* = 0.02) and *HER3*(*R* = 0.33; *p* = 0.03) in a cohort of primary PTCs. In addition, by analyzing TCGA RNA-Seq dataset, we found a positive relationship between *EHF* expression and the expression of *HER2* (*R* = 0.18; *P* < 0.0001) and *HER3* (*R* = 0.63; *P* < 0.0001) in a large cohort of PTCs (Figure 5B). The similar results were also observed in gastric cancers from TCGA database (Supplementary Figure S2). Given that mutation and amplification may affect the expression of *EGFR*, *HER2*, *HER3* and *HER4*, we excluded the cases harboring the above mutation or amplification, and re-analyzed the relationship between *EHF* expression and the expression of HER family using TCGA database (Supplementary Figure S3). The results were consistent with the findings in Supplementary Figure S1. Next, we attempted to evaluate the impact of EHF depletion on the expression of these genes in thyroid cancer cells. Our data indicated that the expression of *HER2* and *HER3*, but not *EGFR* was significantly reduced upon EHF knockdown in both BCPAP and C643 cells compared with the controls (Figure 5C, left panels). *HER4* expression was not detectable in these two cell lines. On the other hand, ectopic expression of EHF in FTC133 and K1 cells significantly increased the expression of *HER2* and *HER3*, but not *EGFR* (Figure 5C, right panels). These findings were further supported by the results of western blot (Figure 5D). Collectively, we suggest that the transcription of *HER2* and *HER3* may be regulated by EHF in thyroid cancer.

**Figure 6:**
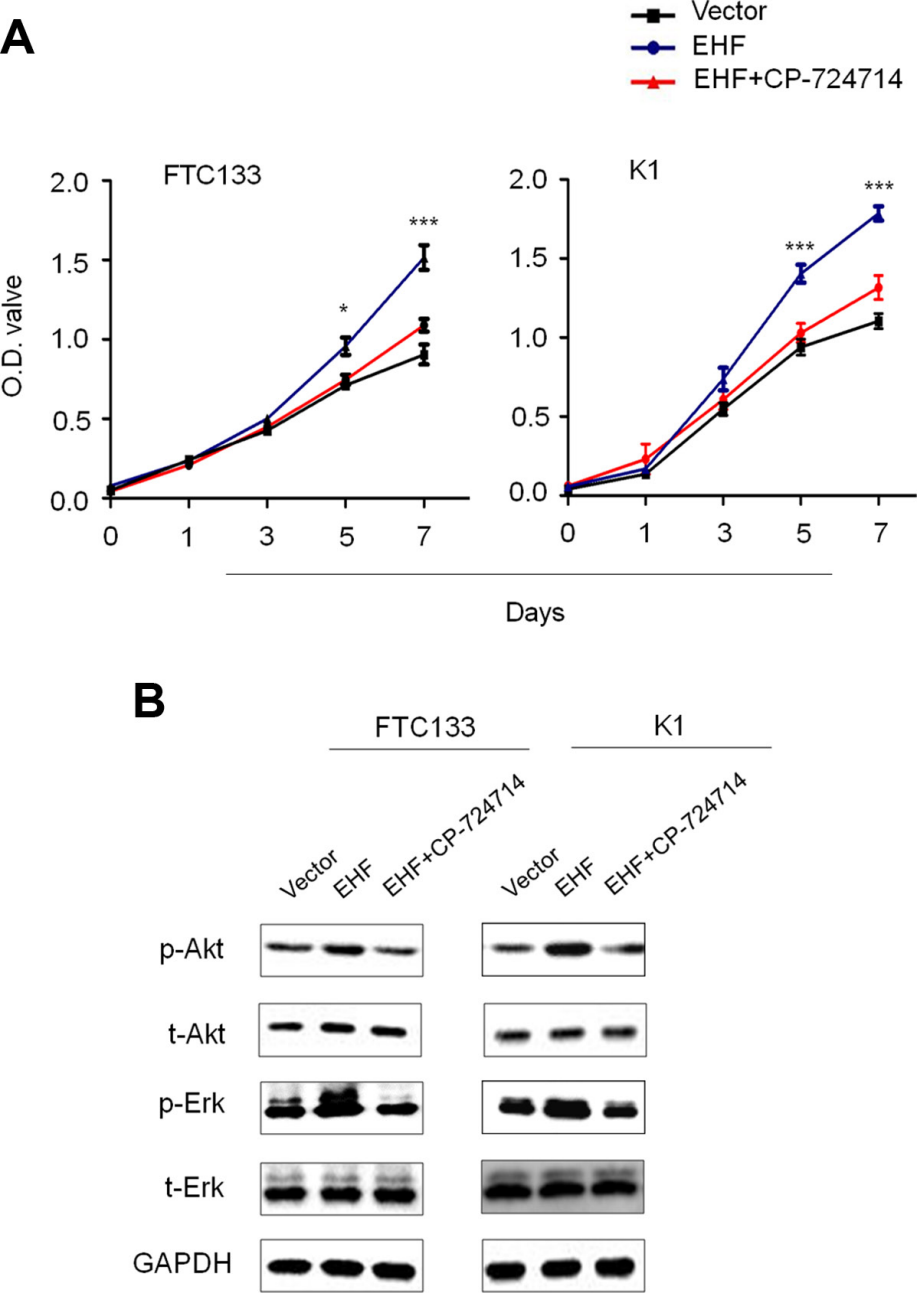
Tumor-promoting effect of EHF through activating HER2 signaling (**A**) The effect of HER2 inhibition by CP-724714 on proliferation-promoting role of EHF in FTC133 and K1 cells was assessed by MTT assay. (**B**) Western blot assay was performed to evaluate the activities of two main downstream pathways of HER2, the PI3K/Akt and MAPK/Erk cascades, upon HER2 inhibition by CP-724714 in FTC133 and K1 cells. GAPDH was used as loading control in western blot assay. Statistically significant differences were indicated: *P < 0.05; ***P < 0.001.

There is a growing body of evidence suggesting overexpression of HER family members leads to the activation of its downstream pathways such as the MAPK/Erk and PI3K/Akt cascades [21]. Thus, we tested the effect of EHF knockdown on the activities of these two pathways. As expected, EHF knockdown and overexpression correspondingly inhibited and promoted the phosphorylation of Erk (p-Erk) and Akt (p-Akt) (Figure 5E and 5F). This result is consistent with the finding of the recent study which has showed that the levels of p-Erk and p-Akt were inhibited by EHF knockdown in ovarian cancer cells [16]. To further determine the oncogenic activity of EHF in thyroid cancer cells through activating HER2/HER3, we used a selective HER2 receptor tyrosine kinase inhibitor CP-724714 to suppress HER2 activation in the indicated cells. As shown in Figure 6A, proliferation-promoting role of EHF in these cells was dramatically attenuated upon HER2 inhibition. In addition, EHF-mediated the activation of two main downstream pathways of HER2, the PI3K/Akt and MAPK/Erk cascades, was reversed by CP-724714 in these cells (Figure 6B).

**Figure 7:**
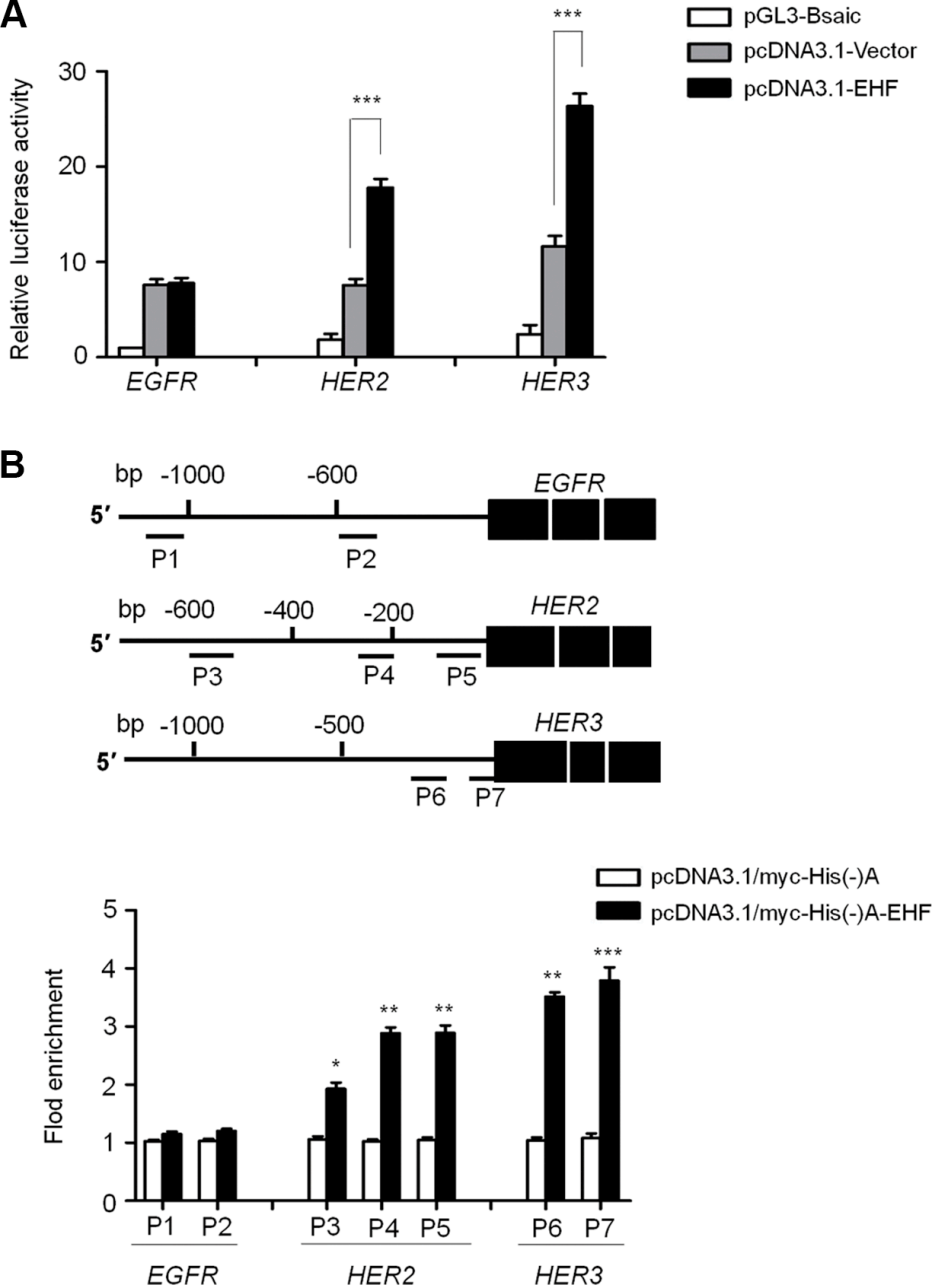
EHF is identified as a new transcription factor for *HER2* and *HER3* in thyroid cancer (**A**) The luciferase reporter gene assay was performed to evaluate the effect of EHF knockdown on promoter activities of *EGFR*, *HER2* and *HER3* in FTC133 cells. The ratio of the Luc/Renilla activity is shown as means ± SE of three independent assays. (**B**) Putative promoter regions of EGFR (−1051/+101), HER2 (−607/+11) and *HER3* (−997/+440) were inserted into the pGL3-Basic to construct the luciferase reporter plasmid pGL3-EGFR-Luc, pGL3-HER2-Luc and pGL3-HER3-Luc (upper panels). P1-P7 represent the regions analyzed by ChIP assays for *EGFR*, *HER2* and *HER3*, respectively. FTC133 cells were transiently transfected with pcDNA3.1/myc-His(−)A-EHF or empty vector, and were subjected to ChIP-qRT-PCR assays using anti-Myc tag antibody. Flod enrichment was shown as means ± SE of three independent assays (lower panels). Statistically significant differences were indicated: *P < 0.05; **P < 0.01; ***P < 0.001.

To further determine whether transcription factor EHF was indeed involved in directly regulating promoter activities of *EGFR*, *HER2* and *HER3*, we cloned their promoters into a pGL3-Basic luciferase plasmid to construct luciferase reporter plasmids including pGL3-EGFR-Luc (−1051/+101), pGL3-HER2-Luc (−607/+11) and pGL3-HER3-Luc (−997/+440). As shown in Figure 7A, ectopic expression of EHF in FTC133 cells was able to significantly increase promoter activities of *HER2* and *HER3* (*P* < 0.001), but not *EGFR*. Next, we attempted to demonstrate whether EHF regulated the activities of *HER2* and *HER3* by directly binding to their promoters. Thus, the ChIP assay was performed in the EHF-transfected FTC133 cells and control cells, followed by qRT-PCR targeting their promoter regions. As shown in Figure 7B, EHF strongly bound to the prompter of *HER2* and *HER3* in FTC133 cells, but not *EGFR*. Three different fragments within *HER2* promoter P3: −604/−484; P4: −274/−155; P5: −147/−37) and two different fragments with *HER3* promoter (P6: −203/−81; P7: −77/+43) were enriched by 2.57- and 3.65-fold on average in pcDNA3.1/myc-His(−)A-EHF-transfected cells compared with vector-transfected cells, respectively. To be consistent with the dual luciferase findings, the ChIP assays further support *HER2* and *HER3* genes as potential targets of EHF in thyroid cancer.

## DISCUSSION

It is clear that ETS factors such as ETS-1 and ETS-2 play a critical role in thyroid cell transformation [22]. However, the role of another member of ETS family, EHF, in thyroid tumorigenesis remains unclear. In this study, we first provided evidences suggesting the oncogenic function of EHF in thyroid cancer. First, *EHF* was frequently upregulated in PTCs compared to matched control samples. Second, EHF knockdown significantly inhibited tumorigenic potential of thyroid cancer cells, whereas EHF overexpression enhanced the growth and motile/invasive ability of cancer cells. Third, EHF was identified to be a new transcription factor of *HER2* and *HER3* in thyroid cancer.

Although *EHF* overexpression has been reported in different cancers, including gastric, ovarian and mammary cancers [15–17, 23], the role and mechanisms of EHF in thyroid tumorigenesis remain totally unknown. We thus investigated its biological role in thyroid cancer cells by a series of *in vitro* and *in vivo* studies. As expected, EHF knockdown exhibited strong growth-inhibitory activity by suppressing cell proliferative and colony forming abilities *in vitro* and tumorigenic potential in nude mice *in vivo*. Moreover, EHF knockdown induced cell cycle arrest and apoptosis, and reduced the ability of cell migration/invasion. Conversely, EHF overexpression significantly promoted cell growth and invasiveness, further suggesting that EHF possesses a strong tumorigenic function in thyroid cancer.

As a member of the ETS family, downstream targets or pathways of EHF in thyroid cancer remain to be identified. It is the fact that there is a conserved ETS-responsive element (GAGGAA) in the proximal *HER2* promoter, and indicated that it is recognized by an ETS-immunoreactive factor in breast cancer cells [24]. However, until now, although more than 10 different ETS transcription factors have been found in human cancers, only a few ETS family members such as Elf-1 [25], PEA3 [26], ESE1 [27, 28] have been reported as potential *HER2* transactivators. By using MatInspector, we found the core GGAA/T motif (ETS binding site, EBS) in the *HER2* and *HER3* promoters. Consistently, we found positive associations of *EHF* expression with expression *HER2* and *HER3* in a cohort of primary PTCs, as supported by the information from TGCA database. In addition, knockdown or ectopic expression of EHF in thyroid cancer cells significantly reduced or increased mRNA expression of *HER2* and *HER3*, respectively. These data suggest that *HER2* and *HER3* may be potential downstream targets of EHF. Indeed, the luciferase reporter gene assays showed that ectopic expression of EHF significantly enhanced promoter activities of *HER2* and *HER3* in thyroid cancer cells, also supported by the ChIP assay. Accordingly, EHF knockdown remarkably inhibited the activity of their downstream signaling pathways such as the MAPK/Erk and PI3K/Akt cascades. These observations suggest that EHF may be a new transcription factor for *HER2* and *HER3* in thyroid cancer by binding to a functional EBS within their promoter, and plays its oncogenic function by activating HER family of receptor tyrosine kinases, contributing to thyroid tumorigenesis.

The previous studies have indicated that EHF acts as an oncosuppressor in prostate cancer and ESCC [11, 13, 14], whereas our data suggest that EHF may have a putative oncogenic function in thyroid cancer. We thus speculate that EHF derived from different types of cells or tissues may play a distinct biological role in tumorigenesis. It is the fact that the DNA consensus sequences determined for the different ETS proteins are very similar, and thus their specific functions are dependent on other factors including interaction with other nuclear factors [29, 30]. ETS proteins interact with other transcription factors to form the complexes and enhance their transcriptional activity. In addition, such interactions of ETS with other cofactors also define the specificity of their target genes and play distinct roles in a tissue-specific manner. It is well known that a common feature of human cancers is aberrant activation of some pathways such as the MAPK/Erk and PI3K/Akt cascades. Usually, aberrant expression of some ETS proteins as downstream effectors of these pathways is frequently found in tumorigenesis. In turn, ETS factors can also affect the activities of these pathways through regulating the transcription of multiple receptor tyrosine kinases including HER2 and HER3 [31]. In addition, posttranslational modifications such as phosphorylation, acetylation, sumoylation, ubiquitinylation and glycosylation directly regulate the activity of ETS factors through affecting their cellular localization, DNA-binding activity, transactivation and stability [32–34].

In summary, we found *EHF* overexpression in PTCs and demonstrated that EHF promotes thyroid cancer cell growth and invasiveness through transcriptionally regulating *HER2* and *HER3*. Altogether, our data define the oncogenic roles of EHF in thyroid tumorigenesis and suggest that EHF have potential therapeutic value for the treatment of thyroid cancer.

## MATERIALS AND METHODS

### Clinical samples

A total of 37 primary PTCs and their matched non-cancerous thyroid tissues were randomly obtained from the First Affiliated Hospital of Xi'an Jiaotong University. None of these patients received any therapeutic intervention before surgery. All of the tissues were histologically examined by two senior pathologists at Department of Pathology of the Hospital based on World Health Organization (WHO) criteria. Informed consent was obtained from each patient before the surgery, and the study protocol was approved by the Institutional Review Board and Human Ethics Committee of the First Affiliated Hospital of Xi'an Jiaotong University.

### RNA extraction and quantitative RT-PCR (qRT-PCR)

Total RNA from the tissues and cell lines were isolated by TRIzol reagent following manufacturer's instruction (Takara Inc., Dalian, China), and cDNA was prepared using PrimeScript RT reagent Kit (Takara Inc., Dalian, China). Quantitative RT-PCR (qRT-PCR) was carried out on a CFX96 Thermal Cycler Dice™ real-time PCR system (Bio-Rad Laboratories, Inc., CA) using SYBR Premix Ex Taq™ (Takara Inc., Dalian, China). The mRNA expression of the indicated genes was normalized to *18S* rRNA cDNA. Each sample was run in triplicate. The primer sequences were presented in Supplementary Table S1.

### Cell culture, short interfering RNA (siRNA) and expression plasmid transfection

Human thyroid cancer cell lines BCPAP, FTC133, K1 were provided by Dr. Haixia Guan (The First Affiliated Hospital of China Medical University, Shenyang, China). C643 was provided by Dr. Lei Ye (Ruijin Hospital, Shanghai, China). Cells were all routinely cultured at 37°C in RPMI 1640 medium with 10% fetal bovine serum (FBS), except for FTC133 that was cultured in DMEM/Ham's F-12 medium (Invitrogen Technologies, Inc, CA). Oligonucleotides of siRNA targeting *EHF* (si-EHF-1 and si-EHF-2) and control siRNA (si-NC) were obtained from GenePharma (Shanghai, China) and the sequences were presented in Supplementary Table S2. Cells were transfected at 70% confluence using Lipofectamine 3000 (Invitrogen, Grand Island, NY), with a final siRNA concentration of 50 nM. Specific oligonucleotides with maximal knockdown efficiency were selected among three different sequences until use. All silencing experiments were performed in three replicates. To construct *EHF* expression plasmid, the full-length open reading frame (ORF) of human EHF with or without stop codon TGA was amplified and then cloned into pcDNA3.1(−)A mammalian expression vector with a Myc-His tag (Invitrogen, Grand Island, NY), which was designated as pcDNA3.1/myc-His(−)A-EHF or pcDNA3.1(−)A-EHF.

### Western blot analysis

The indicated cells were lysed in RIPA buffer containing protease inhibitors and phosphatase inhibitor Cocktail (Sigma, Mo, USA). Supernatants were collected and subjected to 10% SDS-PAGE, and transferred onto PVDF membranes (Roche Diagnostics, Mannheim, Germany). The membranes were then incubated overnight with primary antibodies. Anti-phospho-Akt^Ser473^, anti-phospho-Erk1/2, anti-total Akt and anti-total Erk were purchased from Bioworld Technology, co, Ltd. Anti-EHF was purchased from Abcam Biotechnology, Inc. Anti-GAPDH was purchased from Abgent, Inc. This was followed by incubation with species-specific HRP-conjugated secondary antibodies from ZSGB-BIO, and antigen-antibody complexes were visualized using the Western Bright ECL detection system (Advansta, CA).

### Cell proliferation, colony formation, cell cycle and apoptosis assays

The procedures for cell proliferation, colony formation, cell cycle and apoptosis assays were similarly performed as described previously [35].

### *In vivo* tumorigenicity

Four- to five-week-old female athymic nude mice were purchased from SLAC laboratory Animal Co., Ltd. (Shanghai, China) and housed in a specific pathogen-free (SPF) environment. The mice were randomly divided into two groups (five mice per group). Tumor xenografts were established by subcutaneously injecting 3×10^6^ C643 cells transfected with the indicated siRNAs into the oxter of nude mice. From day 3 post-injection, tumor size was measured every 2 days. Tumor volumes were calculated by the formula (length × width^2^ × 0.5). At the end of the experiment, mice were sacrificed, and tumors were harvested and weighted. Tumors obtained from representative animals were embedded in paraffin, sectioned at 5 μm, and stained with H&E. Ki-67 staining was used to evaluate cell proliferation. Moreover, the TUNEL assay was perfomed to evaluate cell apoptosis *in vivo* using the TUNEL Andy Fluor™ 594 Apoptosis Detection Kit (GeneCopoeia, Inc.) according to the manufacturer's instructions. All experimental procedures were approved by the Animal Ethics Committee of Xi'an Jiaotong University.

### Cell migration and invasion assays

The procedures of cell migration and invasion assays were similarly performed as described previously [35].

### Luciferase reporter assays

The promoter regions of *HER2* and *HER3* genes were cloned into the pGL3-Basic luciferase vector (Promega Corp., Madison, WI) to produce the luciferase reporter plasmids pGL3-HER2-Luc and pGL3-HER3-Luc. All of the constructs were verified by Sanger sequencing. The primers for plasmid constructs were presented in Supplementary Table S3. Next, to test promoter activity of *HER2* and *HER*3 genes regulated by EHF, FTC133 cells were transfected with pcDNA3.1(−)A-EHF or empty vector in 6-well plates and were cotransfected with pGL3-HER2-Luc, pGL3-HER3-Luc, and pRL-TK plasmids (Promega Corp., Madison, WI) using Lipofectamine 3000 (Invitrogen, Grand Island, NY). The pRL-TK plasmid, containing Renilla luciferase, was commonly used to normalize transfection efficiency. Cells were harvested 36 h post-transfection, and luciferase activities were analyzed on EnSpire^®^ Multimode Plate Reader (PerkinElmer, Waltham, MA) using the dual-luciferase reporter assay system (Promega Corp., Madison, WI) according to the manufacturer's instructions. Data were expressed as relative luciferase activity (Firefly luciferase activity/Renilla luciferase activity). Each experiment was performed in triplicate.

### Chromatin immunoprecipitation (ChIP) assay

The ChIP assay was performed to test EHF binding to its target DNA using the Pierce™ Magnetic ChIP Kit (Pierce Biotechnology, Rockford, IL) according to the manufacturer's instructions. Briefly, FTC133 cells were transfected with pcDNA3.1/myc-His(−)A-EHF and empty vector. After 2 days, the indicated cells (1 × 10^7^cells) were incubated with 1% formaldehyde to cross-link the DNA-protein complexes and the cross-linking reaction was stopped by the addition of glycine. The cells were then collected and lysed with SDS lysis buffer (1% SDS, 10 mM EDTA, 50 mM Tris, pH 8.1) containing protease inhibitors. Lysate was sonicated to shear the DNA to lengths between 200 and 1000 bp. Next, 10% of the chromatin from each lysate was saved as an input control. The remaining chromatin was immunoprecipitated by using mouse monoclonal anti-Myc tag, clone 4A6 antibody (Millipore, Temecula, CA). The same amount of non-specific IgG was used as negative control. Immunoprecipitated protein DNA complex was then captured with ChIP Grade Protein A/G Magnetic Beads. The DNA was extracted using standard phenol/chloroform protocol. These DNA fragments were then used as templates for qRT-PCR analysis using the primers presented in Supplementary Table S4, and the data were normalized by respective 5% input. Each experiment was performed in triplicate.

### Statistical analysis

The data are expressed as mean ± standard deviation (SD) or standard error (SE) of the mean as indicated. Statistical significance of differences between the results was assessed using a standard 2-tailed *t* test and Mann–Whitney *U* test, conducted using SPSS statistical package (16.0, Chicago, IL). *P* < 0.05 was considered statistically significant.

## SUPPLEMENTARY MATERIALS TABLES AND FIGURES


