# Supplementary material for: Increased expression of EHF contributes to thyroid tumorigenesis through transcriptionally regulating HER2 and HER3
Source: Oncotarget. 2016 Aug 9;7(36):57978–90. doi: 10.18632/oncotarget.11154 (PMC5295405; doi:10.18632/oncotarget.11154)
Supplement: Supplementary file 1 [file oncotarget-07-57978-s001.pdf]

Increased expression of EHF contributes to thyroid tumorigenesis through transcriptionally regulating *HER2* and *HER3*

Supplementary Materials

Supplementary Table S1: qRT-PCR primers used in this study

Genebank (ID)	Genes	Forward primer (5′-3′)	Reverse primer (5′-3′)	Product length (bp)
NM_012153.5	*EHF*	TGATTCTGGAAGGAGGTGGT	ATGTCGAACTCTTGGAAA GGG	238
NM_005228.3	*EGFR*	GGGCTCTGGAGGAAAAGAAA	AAATTCCCAAGGACCAC CTC	127
NM_001005862.2	*HER2*	ATCAACTGCACCCACTCCTG	TGATGAGGATCCCAAAGA CCAC	145
NM_001005915.1	*HER3*	AGTCATGAGGGCGAACGAC	TCACACTCAGGCCATTC AGA	119
NM_001042599.1	*HER4*	ACGGGATCTGAGACTTCCAA	TTATTCTCCGTTCCTGCACA	127
R_003286.2	*18S*	CGCCGCTAGAGGTGAAATTC	CTTTCGCTCTGGTCCGTCTT	52

Supplementary Table S2: The sequences of siRNAs used in this study

si-RNAs	Sequences (5′-3′)
si-EHF-1 (sense)	GCCAGUGGCAUGAAAUUCATT
si-EHF-1 (antisense)	UGAAUUUCAUGCCACUGGCTT
si-EHF-2 (sense)	CAGCCGAGCUAUGAGAUAUTT
si-EHF-2 (antisense)	AUAUCUCAUAGCUCGGCUGTT
si-NC (sense)	UUCUCCGAACGUGUCACGUTT
si-NC (antisense)	ACGUGACACGUUCGGAGAATT

Supplementary Table S3: The primers used for luciferase reporter plasmid constructs in this study

Plasmids	Position	Forward primer (5′–3′)	Reverse primer (5′–3′)	Restriction sites
pGL3-EGFR-Luc	–1051/+101	CGGGGTACCTCTAAAAG CACCTCCACGGC	CCCAAGCTTGACACGCCC TTACCTTTCTTT	Kpn1& HindIII
pGL3-HER2-Luc	–607/+11	CGGGGTACCAAGTCCTT TCGATGTGACTGTC	CCGCTCGAGCTGGTTTCT CCGGTCCCAAT	Kpn1& Xho1
pGL3-HER3-Luc	–997/+440	CGGGGTACCAGGTTGCA TATCAATAGGGAGC	CCCAAGCTTGACTCCGCA GAGGGTGAAG	KpnI & HindIII

Supplementary Table S4: qRT-PCR primers used for the ChIP assay in this study

Genes	Position	Forward primer (5′-3′)	Reverse primer (5′-3′)	Product length (bp)
EGFR	P1: –1212/–1090	CTGCAGGAGAAGGAA CAGTGG	AGAAATGCCAGGGAAACTCG	122
	P2: –594/–482	GCACAGATTTGGCTC GACCT	GGGTGCCCTGAGGAGTTAATTT	112
HER2	P3: –604/–484	TCCTTTCGATGTGAC TGTCTCC	TGTGTTTACCTTGTGGCTTCC	121
	P4: –274/–155	TGCATTTAGGGATTC TCCGA	ACTCCCAGCTTCACTTTCTC	120
	P5: –147/–37	CCCAGACTTGTTGG AATGCAG	ATTCTTATACTTCCTCAAGCAGCC	111
HER3	P6: –203/–81	TTCGAGTCTGGGAGA AACTGAG	TAGCCGGTTGGTTCACTTGG	123
	P7: –77/+43	GAGTTGAGTGATTTG GTTAATGGG	GAGGTCGAGATTCCGAAAGC	120

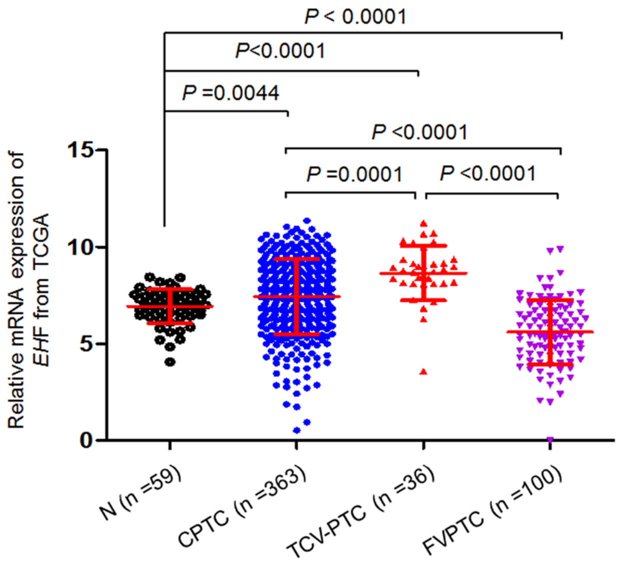

Supplementary Figure S1: *EHF* expression in normal thyroid tissues (N) and different subtypes of PTCs in The Cancer Genome Atlas (TCGA) dataset. Horizontal lines indicate the median and interquartile range. CPTC, conventional PTC; FVPTC, follicular variant of PTC; TCV-PTC, tall-cell variant of PTC.

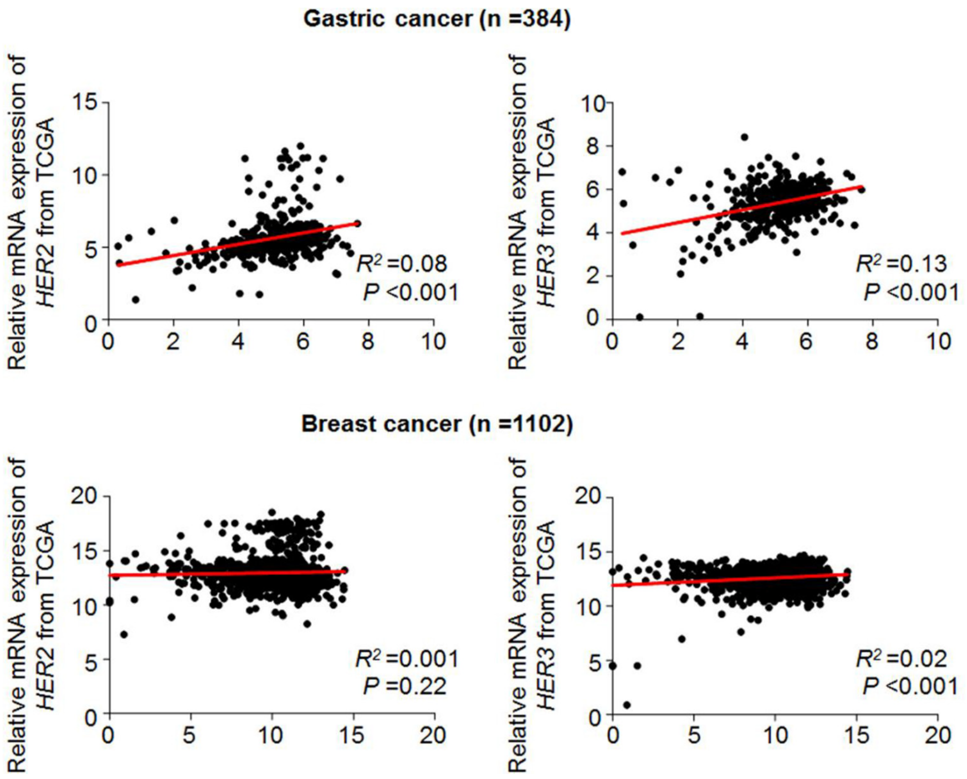

Supplementary Figure S2: The relationships between with mRNA expression of *EHF* and HER receptors (EGFR, HER2, HER3 and HER4) in gastric and breast cancers from TCGA cohort.

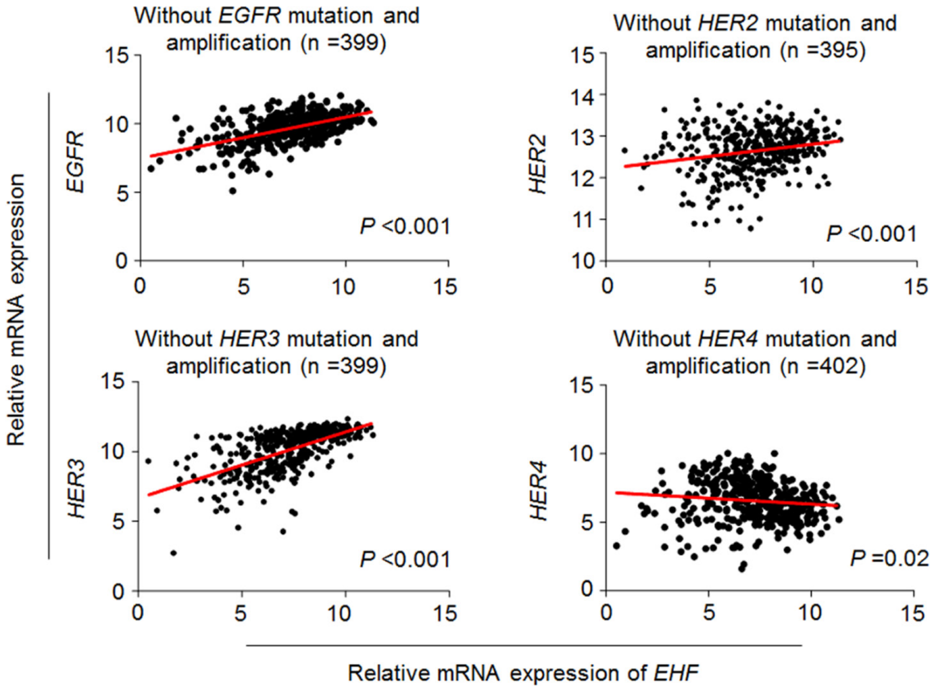

Supplementary Figure S3: The relationship of mRNA expression of *EHF* and HER receptors in a cohort of PTCs without mutations or amplification of *EGFR*, *HER2*, *HER3* and *HER4* from TCGA dataset.
